# EFNA5 as an Oocyte-Derived Factor Enhances Developmental Competence by Modulating Oxidative Stress, Inflammation, and Apoptosis During In Vitro Maturation

**DOI:** 10.3390/antiox14121476

**Published:** 2025-12-09

**Authors:** Xingyuan Liu, Jian Cui, Yubing Wang, Jia Hao, Yingjie Wu, Yinjuan Wang, Lei An, Jianhui Tian, Guangyin Xi

**Affiliations:** College of Animal Science and Technology, China Agricultural University, Beijing 100193, China; 19806395675@163.com (X.L.); 18801364796@163.com (J.C.); wangyubing911@163.com (Y.W.); 17630751829@163.com (J.H.); wuyingjie@cau.edu.cn (Y.W.); wangyinjuan@cau.edu.cn (Y.W.); anleim@cau.edu.cn (L.A.)

**Keywords:** in vitro maturation, oocyte-derived factors, EFNA5, oxidative stress, inflammation, NRF2, NF-κB

## Abstract

In vitro maturation (IVM) of oocytes remains suboptimal due to oxidative stress and disrupted cumulus–oocyte communication. Oocyte-derived factors (ODFs) are key mediators of this crosstalk and crucial for oocyte competence. Here, we provide systematic evidence that ephrin-A5 (EFNA5) is an oocyte-derived membrane ligand capable of regulating oocyte quality during IVM. Cross-species transcriptomic analysis revealed that EFNA5 is stably enriched in mammalian oocytes but markedly reduced in in vitro-matured oocytes compared with in vivo counterparts. Using the ovine IVM model, supplementation with recombinant EFNA5 significantly improved blastocyst formation, increased total cell numbers, and reduced apoptosis. Mechanistically, EFNA5 promoted cumulus–oocyte complex expansion, reduced reactive oxygen species accumulation, activated NRF2-dependent antioxidant signaling, and suppressed NF-κB-driven inflammation. RNA-seq and functional validation further confirmed that EFNA5 enhanced redox homeostasis and decreased DNA damage, collectively improving oocyte developmental potential. These findings establish EFNA5 as a novel and conserved ODF that alleviates oxidative and inflammatory stress to enhance oocyte quality and embryo development, providing mechanistic insight and a potential strategy for improving assisted reproductive technologies.

## 1. Introduction

In vitro maturation (IVM) of oocytes represents a central technology in both animal in vitro embryo production (IVEP) systems and contemporary human assisted reproductive technologies (ART) [1,2,3]. Continued improvements in IVM efficiency hold the potential to further optimize ART, offering significant advancements for the treatment of human infertility and the enhancement of livestock breeding. However, compared with oocytes matured in vivo (IVO), the efficiency of IVM remains suboptimal, as oocytes matured in vitro often exhibit reduced developmental competence [4,5]. A major limitation lies in the inability of current culture systems to fully recapitulate the ovarian microenvironment, leading to excessive oxidative stress, mitochondrial dysfunction, and elevated inflammatory responses [2,6]. These adverse conditions ultimately compromise both nuclear and cytoplasmic maturation of oocytes, resulting in decreased fertilization rates and blastocyst formation [4,7]. Consequently, pinpointing paracrine or autocrine factors that reshape the oocyte IVM microenvironment to reduce oxidative stress, suppress apoptosis, and fortify cumulus–oocyte crosstalk is indispensable for unlocking high-quality oocyte production and the future of ART.

Oocyte-derived factors (ODFs) are the principal architects of mammalian folliculogenesis and oocyte competence. Increasing evidence indicates that oocytes are not merely passive recipients of cumulus cell support but actively regulate cumulus cell function and their own developmental competence through the secretion of specific growth factors, thereby maintaining oocyte quality and the homeostasis of the follicular microenvironment [8]. Canonical ODFs include growth differentiation factor 9 (GDF9) and bone morphogenetic protein 15 (BMP15), two TGF-β superfamily ligands, which promote granulosa cell proliferation and differentiation, induce cumulus cell expansion, and enhance oocyte developmental competence by facilitating bidirectional cumulus–oocyte signaling [9,10]. This core network is fine-tuned by ancillary ODFs such as fibroblast growth factor 8 (FGF8), KIT ligand (KITL), and R-spondin-2 (RSPO2), which calibrate granulosa metabolism, sustain oocyte survival, and safeguard mitochondrial function [11,12]. Together, these oocyte-borne signals converge with granulosa-derived factors to sculpt a microenvironment that licenses high-quality maturation and primes the oocytes for robust embryonic development, offering actionable targets for the rational design of IVM and embryo culture systems.

In recent years, intercellular communication has gained increasing attention for its role in regulating follicular microenvironment stability and oocyte developmental competence [13]. The Ephrin (EFN) family, comprising glycosylphosphatidylinositol (GPI)-anchored or transmembrane ligands, mediates cell adhesion, migration, and tissue morphogenesis through interactions with Eph receptor tyrosine kinases [14]. While extensive research has established their functions in neural development [15], angiogenesis, and tumorigenesis, emerging evidence suggests that ephrins may also participate in reproductive processes. Within the ovary, studies using gene knockout mouse models have demonstrated that ephrin/Eph signaling participates in folliculogenesis and the ovulatory cascade [16,17], yet its specific functions during oocyte maturation remain largely unexplored [16]. Additionally, analyses of published datasets detect EFNA5 expression in germ cells across multiple species, collectively suggesting that EFNA5 may play some relatively conservative roles in reproduction [18,19,20].

In this study, to dissect the regulatory role and underlying mechanism of EFNA5 in oocyte maturation, we exploited an ovine IVM model that is both agriculturally relevant and translationally informative for human reproductive medicine. We first mapped the spatiotemporal expression of EFNA5 and its cognate receptor EPHA4 (selected due to its highest expression among EphA receptors in ovine oocytes) in ovine ovaries, then systematically evaluated the impact of exogenous recombinant EFNA5 on oocyte meiotic progression, blastocyst yield, and the attendant molecular circuitry. Our results showed that EFNA5, secreted by the oocyte itself, elevates nuclear and cytoplasmic maturation and augments subsequent embryonic potential by simultaneously reducing oxidative stress, curbing inflammatory signaling, and reinforcing bidirectional cumulus–oocyte communication. This study delivers the first functional evidence that EFNA5 operates as a novel oocyte-derived factor and provides a mechanistic framework for enhancing oocyte IVM efficiency while offering translational insights for human ART.

## 2. Materials and Methods

### 2.1. Chemicals

All chemicals and reagents, unless otherwise specified, were purchased from Sigma-Aldrich (St. Louis, MO, USA). Goat anti-rat IgG (H+L) cross-adsorbed secondary antibody and DAPI solution were obtained from Invitrogen (Carlsbad, CA, USA). HRP-conjugated Affinipure goat anti-rabbit IgG (H+L) was purchased from Proteintech (Beijing, China).

### 2.2. Oocyte Collection and In Vitro Maturation

Sheep ovaries were collected in multiple independent batches from a local slaughterhouse (Ulanqab, Inner Mongolia, China). Each batch comprised at least 20 pairs of ovaries, and all experiments were repeated at least three times. Following collection, they were transported to the laboratory within 2 h in 0.9% saline that was maintained at 36 °C and contained 100 IU/mL penicillin. Upon arrival, ovaries were rinsed thoroughly with pre-warmed saline, and follicular fluid from 3–8 mm follicles was aspirated using an 18-gauge needle. Cumulus–oocyte complexes (COCs) surrounded by at least three layers of compact cumulus cells were selected under a stereomicroscope. In some experiments, germinal vesicle (GV) oocytes were obtained by treating COCs with 0.3% hyaluronidase (Sigma-Aldrich, St. Louis, MO, USA) followed by gentle pipetting to remove surrounding cumulus cells. For in vitro maturation (IVM), COCs or GV oocytes were cultured in TCM-199 medium (Thermo Fisher Scientific, Waltham, MA, USA) supplemented with 10 µg/mL FSH, 10 µg/mL LH, 1 µg/mL 17β-estradiol, 100 µg/mL L-glutamine, 10% (*v*/*v*) fetal bovine serum (FBS; Gibco, Thermo Fisher Scientific, Waltham, MA, USA), and 1% (*v*/*v*) penicillin–streptomycin (Sigma-Aldrich, St. Louis, MO, USA). Cultures were maintained at 38.5 °C under 5% CO_2_ in humidified air for 24 h. The efficiency of IVM was assessed by polar body extrusion (PBE).

Recombinant human ephrin-A5 (EFNA5; ProSpec-Tany TechnoGene Ltd., Rehovot, Israel; Cat. PRO-2327) was reconstituted in 0.1% BSA in PBS according to the manufacturer’s instructions, sterile-filtered (0.22 µm), aliquoted, and stored at −80 °C to avoid repeated freeze–thaw cycles. For supplementation experiments, the IVM medium was supplemented with EFNA5 at final concentrations of 0, 10, 50, or 100 ng/mL. Vehicle controls received equal volumes of the reconstitution buffer (0.1% BSA/PBS) diluted in IVM medium.

### 2.3. In Vitro Fertilization and Embryo Culture

Following IVM, cumulus cells were removed from COCs via three sequential washes in hyaluronidase-supplemented (0.2% *w*/*v*) synthetic oviductal fluid (SOF). The denuded oocytes were then transferred to IVF medium, which consisted of SOF supplemented with 2% estrous sheep serum, 3 mg/mL BSA, 6 IU/mL heparin, and 50 IU/mL gentamicin. For sperm preparation, frozen straws were thawed in a 39 °C water bath for 1 min. The semen was diluted in pre-equilibrated IVF medium, and motile spermatozoa were isolated by a 30 min swim-up procedure at 38.5 °C under 5% CO_2_ in humid air. The resulting motile fraction was collected and centrifuged at 200× *g* for 5 min, and the pellet was resuspended in IVF medium. The sperm concentration was measured with a hemocytometer and subsequently adjusted to a final concentration of 1 × 10^6^ spermatozoa/mL. Oocytes were co-incubated with spermatozoa for 20 h at 38.5 °C, 5% CO_2_, maximum humidity. Presumptive zygotes were washed three times in IVC medium and cultured in groups of 25–30 in 50 µL droplets of SOF supplemented with 1% (*v*/*v*) BME-essential amino acids, 1% (*v*/*v*) MEM-nonessential amino acids, 1 mM l-glutamine and 3 mg/mL BSA under 38.5 °C, 88% N_2_, 6% CO_2_ and 6% O_2_. Only oocytes that had extruded the first polar body (indicative of maturation to MII) were selected for IVF. After co-incubation with spermatozoa, presumptive zygotes were identified by the presence of a second polar body and two pronuclei under a stereomicroscope. Only these zygotes were selected for subsequent embryo culture, and cleavage and blastocyst rates were recorded at 48 h and on day 6 post-IVF, respectively.

### 2.4. Evaluation of COCs’ Expansion

For each biological replicate, COCs were randomly assigned to four IVM groups receiving 0, 10, 50, or 100 ng/mL EFNA5, with 30–50 COCs per group. After 24 h of IVM, cumulus expansion was evaluated according to the criteria adapted from Vanderhyden et al. [21]. and scored on a scale of 1–3: 1, partial expansion restricted to the outermost cumulus cell layers; 2, expansion of all cumulus cell layers except the corona radiata; 3, complete expansion of all cumulus cells, including the corona radiata.

### 2.5. Detection of Apoptosis

To determine the anti-apoptotic effect of EFNA5 during IVM and its persistence into embryonic development, apoptosis was assessed in both cumulus–oocyte complexes (COCs) and the resulting blastocysts. Briefly, COCs were collected immediately after the 24 h IVM period to evaluate the direct impact of EFNA5 on the maturation microenvironment. Subsequently, blastocysts derived from these oocytes were analyzed on day 6 to assess the long-term protective effect on embryo quality. Apoptosis was assessed using a one-step TUNEL apoptosis assay kit (Beyotime, Shanghai, China) following the manufacturer’s protocol. Briefly, COCs and blastocysts were washed in PBS, fixed in 4% paraformaldehyde (PFA) for 1 h at room temperature, and permeabilized with 1% Triton X-100 for 5 min. Samples were then incubated with freshly prepared TUNEL reaction mixture for 1 h at room temperature in the dark, followed by nuclear counterstaining with DAPI. The number of apoptotic cells was quantified by counting TUNEL-positive nuclei, and the total cell number was determined based on DAPI staining. Fluorescence signals associated with apoptosis in COCs and blastocysts were captured using a fluorescence microscope. The apoptotic signals were quantified by counting the fluorescent green spots corresponding to the stained regions.

### 2.6. Measurement of Intracellular Reactive Oxygen Species (ROS) and Glutathione (GSH) Levels

Intracellular ROS and GSH levels were evaluated using DCFH-DA (Beyotime, Shanghai, China) and CMF2HC (MCE, Monmouth Junction, NJ, USA), respectively. Briefly, cumulus cells were removed by treatment with IVM medium containing 0.1% (*w*/*v*) hyaluronidase, and denuded oocytes were washed thoroughly in PBS. Oocytes were then incubated with the respective fluorescent probes at 37 °C for 20 min in the dark. After washing, fluorescence signals within the oocytes were captured using a fluorescence microscope, and the relative fluorescence intensity for each oocyte was quantified using ImageJ software (version 1.53c; National Institutes of Health, Bethesda, MD, USA).

### 2.7. Lipid Peroxidation

Lipid peroxidation in oocytes was assessed using the ratiometric probe C11-BODIPY 581/591 (Invitrogen, Carlsbad, CA, USA). Briefly, cumulus cells were removed by treatment with IVM medium containing 0.1% (*w*/*v*) hyaluronidase, and denuded oocytes were washed thoroughly in PBS containing 0.1% (*w*/*v*) polyvinyl alcohol (PVA). Oocytes were incubated live with 2 µM C11-BODIPY in IVM medium at 37 °C for 30 min in the dark, followed by three washes in PBS-PVA. Fluorescence was captured immediately on a fluorescence microscope using appropriate filter sets: reduced (non-oxidized) C11-BODIPY was recorded in the red channel and oxidized C11-BODIPY in the green channel. Fluorescence signals within the oocytes were captured using a fluorescence microscope, and the relative fluorescence intensity for each oocyte was quantified using ImageJ software (version 1.53c), and lipid peroxidation was expressed as the oxidized/reduced ratio (green/red), as specified in figure legends. Results are presented as mean ± SEM on a per-oocyte basis.

### 2.8. γH2AX Staining

DNA damage in oocytes was assessed by γH2AX immunofluorescence. Denuded oocytes were fixed in 4% paraformaldehyde (PFA) at 4 °C for 1 h, permeabilized with 0.5% Triton X-100 for 30 min at room temperature, and blocked in PBS containing 1% (*w*/*v*) BSA for 1 h. Samples were incubated overnight at 4 °C with an anti-γH2AX primary antibody (1:200; Beyotime, Shanghai, China), followed by Alexa Fluor 488-conjugated goat anti-rabbit IgG secondary antibody (1:500; Invitrogen, Carlsbad, CA, USA) for 1 h at room temperature in the dark. Nuclei were counterstained with DAPI (1 µg/mL; Invitrogen, Carlsbad, CA, USA), and fluorescence signals were observed using a laser scanning confocal microscope. Fluorescence signals within the DNA region of the oocytes were captured using a fluorescence microscope and results were expressed as mean ± SEM.

All images were acquired using a stereomicroscope (Zeiss Stemi 508; Zeiss, Oberkochen, Germany) and a fluorescence microscope (SMZ-645; Nikon, Tokyo, Japan) equipped with a digital camera. All samples were captured using identical exposure and gain settings within each experiment.

### 2.9. Cortical Granule Staining

Cortical granule (CG) distribution in oocytes was assessed using FITC-conjugated lectin staining. Denuded oocytes were fixed in 4% paraformaldehyde (PFA) at 4 °C for 30 min, permeabilized with 0.1% Triton X-100 in PBS for 1 h at room temperature, and blocked with PBS containing 1% (*w*/*v*) BSA for 1 h. Samples were incubated overnight at 4 °C in the dark with FITC-conjugated Lens culinaris agglutinin (LCA; 1:300; Thermo Fisher Scientific, L32475, Carlsbad, CA, USA). After three washes in PBS, nuclei were counterstained with DAPI (1 µg/mL; Invitrogen, Carlsbad, CA, USA) for 5 min. Oocytes were mounted on glass slides, and CG distribution patterns were observed using a laser scanning confocal microscope. Oocytes were mounted on glass slides, and CG distribution patterns were observed using a laser scanning confocal microscope.

### 2.10. Immunofluorescence Localization of EFNA5 and EPHA4

Immunofluorescence staining was performed to determine the localization of EFNA5 and its receptor EPHA4 in oocytes and cumulus cells. Samples were fixed in 4% paraformaldehyde (PFA) at 4 °C for 30 min, permeabilized with 0.1% Triton X-100 in PBS for 15 min at room temperature, and blocked with 1% BSA in PBS for 1 h. Cells were incubated overnight at 4 °C with primary antibodies against EFNA5 (1:100; ABMART, TP72055, Shanghai, China) and EPHA4 (1:100; Proteintech, 21875-1-AP, Wuhan, China). After three washes in PBS, samples were incubated with Alexa Fluor 488-conjugated secondary antibodies (1:1000; Invitrogen, Carlsbad, CA, USA) for 1 h at room temperature in the dark. Nuclei were counterstained with DAPI (1 µg/mL; Invitrogen, Carlsbad, CA, USA) for 5 min. EFNA5 and EPHA4 localization was visualized using a laser scanning confocal microscope. Oocytes were mounted on glass slides, and CG distribution patterns were observed using a laser scanning confocal microscope.

### 2.11. Transcriptome Sequencing Analysis

To assess EFNA5-dependent transcriptional changes, MII oocytes and cumulus cells were harvested after 24 h of IVM from control and EFNA5-treated groups for RNA-seq. Only morphologically normal oocytes (extruded first polar body and homogeneous cytoplasm) were selected, with 5–6 oocytes per sample and at least three biological replicates per group. Total RNA was extracted using a single-cell full-length mRNA amplification kit (i-SingleCell, Cat. No. N712; Vazyme, Nanjing, China). Libraries were prepared with a modified Smart-seq2 protocol using the TePrep™ DNA Library Prep Kit V2 (Azenta Life Sciences, Burlington, MA, USA), and library quality was assessed using a Qubit 4.0 Fluorometer and a Fragment Analyzer. Cross-species oocyte RNA-seq datasets from multiple species were obtained from the NCBI Gene Expression Omnibus (GEO; accessions: GSE158539 [22], GSE165546 [23], GSE233232 [24], GSE148022 [18], GSE95477 [25], GSE119906 [26], GSE61717 [27], GSE160334 [28]).

Sequencing was conducted on an Illumina NovaSeq™ X Plus platform (Illumina, San Diego, CA, USA) to generate 150 bp paired-end reads. After adapter trimming and quality control with Cutadapt (v2.10) and Trim Galore (v0.6.5), the clean reads were aligned to the ovine genome (Oar_v3.1, Ensembl 106) using HISAT2 (v2.2.1). Transcript abundance was estimated by RSEM (v1.3.3) and expressed as TPM. Differential expression analysis was performed with DESeq2 (version 1.38.0), applying thresholds of |log_2_FC| > 1.5 and adjusted *p*-value < 0.05. Functional enrichment analysis, including Gene Ontology (GO) and KEGG pathways, was carried out using clusterProfiler (v4.0.5), while gene set enrichment analysis (GSEA) was implemented with GSEA v4z.

### 2.12. RNA Extraction and Quantitative Real-Time PCR (qRT-PCR)

Total RNA was isolated from all samples using a commercial total RNA kit (Tiangen, Beijing, China) and subsequently reverse-transcribed into complementary DNA (cDNA) using a HiScript III RT SuperMix kit (Vazyme, Nanjing, China). Quantitative real-time PCR (qPCR) was carried out in a 20 µL reaction system containing 10 µL of SYBR Premix Ex Taq II (Takara, Dalian, China), 2 µL of cDNA template, 1 µL of each forward and reverse primer (10 µM), and 6 µL of nuclease-free water. The amplification protocol consisted of an initial denaturation at 95 °C for 30 s, followed by 40 cycles of 95 °C for 5 s, 60 °C for 30 s, and 72 °C for 30 s. The ACTB gene was employed as the endogenous reference for normalization, and the relative expression levels of target genes were determined using the comparative 2^−ΔCt^ method. All primer sequences used in this study are listed in Supplementary Table S1.

### 2.13. Protein Extraction and Western Blot Analysis

Oocytes, cumulus cells, or COCs from control and EFNA5-treated groups were lysed in RIPA buffer with protease inhibitors (Beyotime, Shanghai, China). Lysates were sonicated and centrifuged at 12,000× *g* for 10 min at 4 °C. Protein concentrations were measured using a BCA Protein Assay Kit (Thermo Fisher, Waltham, MA, USA). Equal amounts of protein (10 μg) were separated by SDS-PAGE and transferred to PVDF membranes (Millipore, Burlington, MA, USA). Membranes were blocked with 5% skim milk for 1 h at room temperature and incubated overnight at 4 °C with primary antibodies. After washing with TBST, membranes were incubated with HRP-conjugated secondary antibodies (1:8000, Jackson, Lansing, MI, USA) for 1 h at room temperature. Protein signals were detected using ECL substrate (Thermo Fisher, Waltham, MA, USA) and imaged on a ChemiDoc imaging system (Bio-Rad Laboratories, Hercules, CA, USA). Band intensities were quantified with ImageJ (version 1.53c), using GAPDH as the internal control.

For clarity of presentation and consistency across figures, some Western blot images were horizontally flipped to align with the order of sample loading described in the figure legends. The original, uncropped scans of all blots with lanes clearly annotated are provided in the Supplementary Information. The orientation does not affect the data interpretation or conclusions.

### 2.14. Statistical Analysis

All experiments included at least three biological replicates. Statistical analyses were performed using GraphPad Prism 8, with data normality and homogeneity of variance assessed by the Shapiro–Wilk test. Between-group differences were analyzed using a two-tailed t-test (for two groups) or one-way ANOVA with Dunnett’s post hoc test (for multiple groups) when parametric assumptions were satisfied. Data are presented as mean ± SEM. Statistical significance is indicated as * *p* < 0.05, ** *p* < 0.01, *** *p* < 0.001, and **** *p* < 0.0001. Different letters in figures indicate significant differences between groups (*p* < 0.05).

## 3. Results

### 3.1. The Expression Pattern of EFNA5 and EPHA4 in Ovine COCs

**Figure 1:**
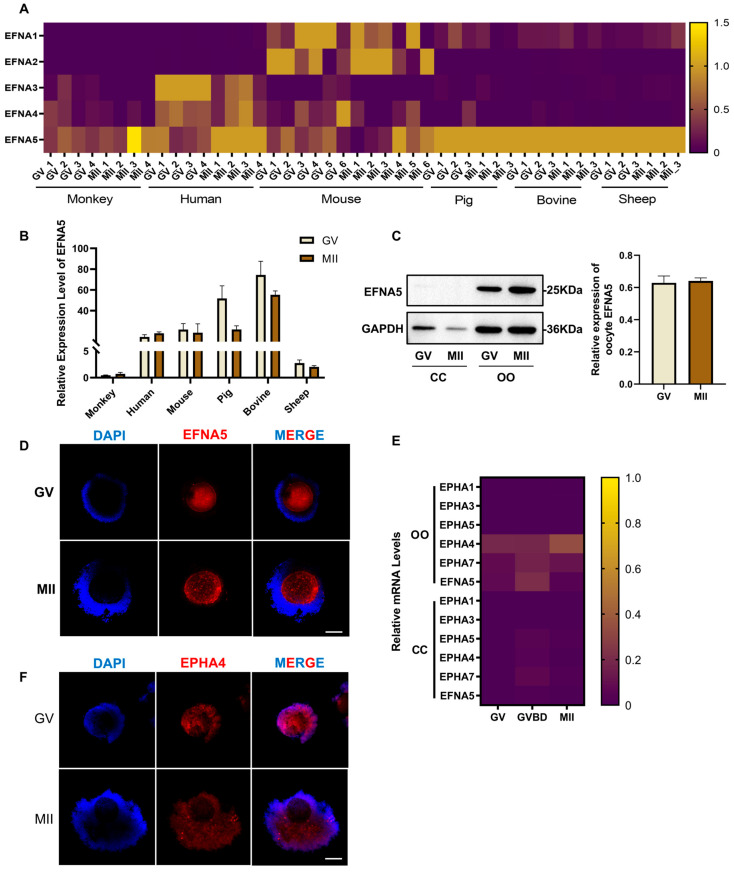
The expression pattern of EFNA5 and EPHA4 in COCs. (**A**) Cross-species heatmaps of EFNA family expression in GV and MII oocytes (row-scaled Z-scores). (**B**) The expression of EFNA5 transcript in GV and MII oocytes from multiple species. (**C**) Western blots of EFNA5 in cumulus cells and oocytes with densitometry normalized to GAPDH. (**D**) Immunofluorescence localization of EFNA5 in COCs (DAPI, blue; EFNA5, red). Scale bars = 50 µm. (**E**) qRT-PCR quantification of EFNA5 and EphA receptors in oocytes and cumulus cells (row-scaled Z-scores). (**F**) Immunofluorescence of EPHA4 in COCs (DAPI, blue; EPHA4, red). Scale bars = 50 µm. Abbreviations: OO, oocyte; CC, cumulus cell; GV, germinal vesicle; MII, metaphase II. All experiments were repeated at least three times. Data are presented as mean ± SEM.

To assess the conservation of EFNA5 in oocyte development, we analyzed transcriptomic datasets from diverse mammalian species, including humans, monkeys, mice, pigs, cattle, and sheep. Although different members of the EFNA family exhibited species-specific expression patterns, *EFNA5* was consistently detected in germinal vesicle (GV) and metaphase II (MII) oocytes across all examined species (Figure 1A). Comparative analysis further revealed that *EFNA5* expression remained relatively stable during the GV-to-MII transition in primates and mice, whereas pronounced changes were observed in livestock species, particularly in ovine oocytes (Figure 1B). These data suggest that EFNA5 may play a relatively conserved role in oocyte development in various species.

To further define the expression pattern of EFNA5, we performed Western blotting on isolated oocytes and cumulus cells at the GV and MII stages. The EFNA5 protein was detected exclusively in oocytes and was undetectable in cumulus cells at both stages (Figure 1C). Immunofluorescence staining further corroborated this result, showing EFNA5 confined to the oocyte with no detectable signal in the surrounding cumulus cells at either stage (Figure 1D). qRT-PCR quantification confirmed that *EFNA5* transcripts were significantly enriched in oocytes relative to cumulus cells, whereas among the Eph receptors assayed, *EPHA4* emerged as the predominantly expressed receptor, with higher levels detected in both cell types (Figure 1E). Immunofluorescence staining provides spatial localization, indicating that EPHA4 shows signals detected in both oocytes and cumulus cells (Figure 1F). Collectively, these results confirm that EFNA5 is an oocyte-derived factor with evolutionarily conserved expression and dynamic regulation during oocyte maturation.

### 3.2. EFNA5 Enhances In Vitro-Matured Oocyte Developmental Potential

**Figure 2:**
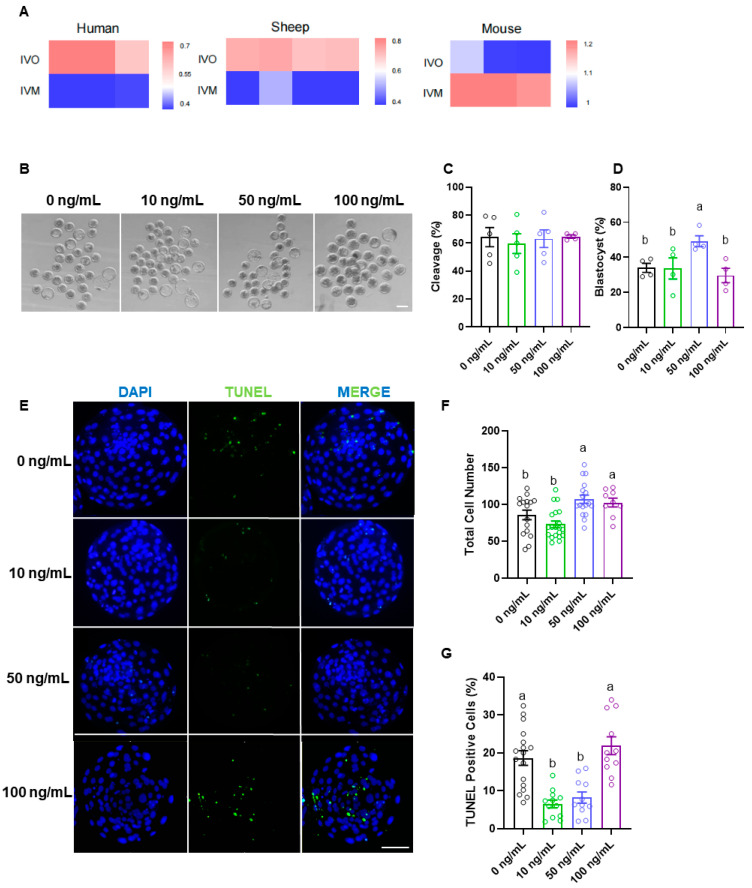
EFNA5 enhances the oocyte developmental competence. (**A**) Cross-species heatmaps comparing EFNA5 expression IVO and IVM oocytes from human, mouse, sheep and mouse (row-scaled Z-scores). (**B**) Representative bright-field images of embryos derived from oocytes matured with 0, 10, 50, or 100 ng/mL recombinant EFNA5. Scale bar = 200 µm. (**C**,**D**) Cleavage rate and blastocyst rate for each group. (**E**) Representative images of blastocysts stained with DAPI (blue) and TUNEL (green). Scale bar = 50 µm. (**F**,**G**) Total cell number and percentage of TUNEL-positive nuclei per blastocyst. Abbreviations: IVO, in vivo maturation; IVM, in vitro maturation. All experiments were repeated at least three times. Data are mean ± SEM. Different letters indicate *p* < 0.05.

We investigated whether oocyte IVM affects the expression of EFNA5. The result revealed that EFNA5 expression in in vitro-matured oocytes was markedly lower than in vivo-matured counterparts in humans and sheep, whereas an opposite trend was observed in mice (Figure 2A). These findings suggest that EFNA5 may represent a key factor lacking in human and sheep in vitro culture systems. Recombinant EFNA5 was supplemented into sheep oocyte IVM medium at concentrations of 0, 10, 50, and 100 ng/mL to assess its functional effects (Figure 2B). EFNA5 treatment did not significantly alter cleavage rates (Figure 2C); however, the blastocyst rate was markedly increased in the 50 ng/mL group (Figure 2D). Blastocyst quality was further evaluated by quantifying total cell numbers and the incidence of apoptosis (Figure 2E). The total blastocyst cell numbers were significantly higher in the 50 ng/mL and 100 ng/mL groups compared with controls (Figure 2F). Consistently, TUNEL assays showed that the proportion of apoptotic cells was also significantly reduced in the 50 ng/mL group (Figure 2G). Collectively, these results indicate that EFNA5 supplementation during IVM enhances oocyte developmental potential, as evidenced by increased blastocyst formation rates and superior blastocyst quality.

### 3.3. EFNA5 Enhances Maturation Status of COCs

**Figure 3:**
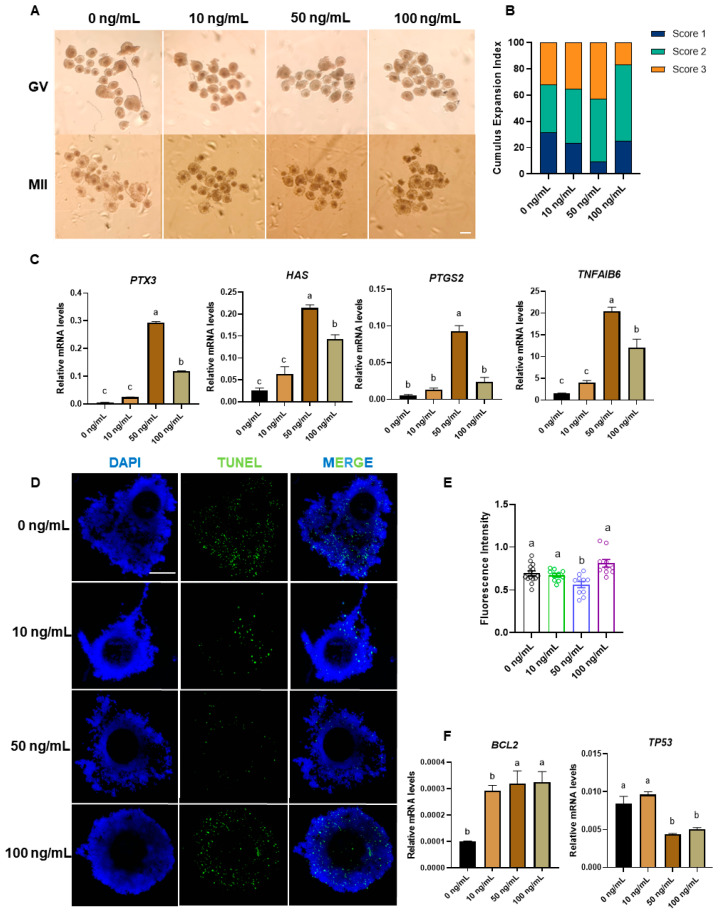
EFNA5 promotes cumulus expansion and suppresses apoptosis in cumulus–oocyte complexes. (**A**) Bright-field images of GV and MII stage COCs matured with 0, 10, 50, or 100 ng/mL EFNA5. Scale bar = 200 µm. (**B**) Distribution of cumulus expansion scores (Score 1, minimal; Score 2, moderate; Score 3, full) under each EFNA5 dose. (**C**) qRT-PCR analysis of cumulus-expansion genes (*PTX3*, *HAS2*, *PTGS2*, *TNFAIP6*) in COCs after IVM. (**D**) Representative images of TUNEL staining in COCs matured with the indicated EFNA5 doses (DAPI, blue; TUNEL, green). Scale bar = 50 µm. (**E**) Quantification of TUNEL fluorescence intensity in COCs. (**F**) qRT-PCR analysis of apoptosis-related genes (*BCL2* and *TP53*) in COCs. All experiments were repeated at least three times. Data are mean ± SEM. Different letters indicate *p* < 0.05.

Because communication between cumulus cells and oocytes is critical for oocyte maturation, we first assessed the effect of EFNA5 supplementation on cumulus expansion during IVM. Morphological evaluation revealed that a greater proportion of COCs achieved the highest expansion grade (Score 3) in the 50 ng/mL EFNA5 group compared with the control (Figure 3A,B). Further analysis revealed that the expression of key genes associated with cumulus cell migration and extracellular matrix remodeling, including pentraxin 3 (PTX3), hyaluronan synthase (HAS), prostaglandin G/H synthase 2 (PTGS2), and tumor necrosis factor alpha-induced protein 6 (TNFAIP6), were significantly upregulated in COCs treated with 50 ng/mL EFNA5 (Figure 3C). These results indicate that EFNA5 can improve the cumulus cell microenvironment, thereby optimizing conditions for oocyte maturation. Because cumulus-cell viability is indispensable for oocyte maturation, we next quantified apoptosis within COCs. TUNEL staining revealed that oocytes in EFNA5-treated groups exhibited a significantly lower proportion of apoptotic cells compared with controls (Figure 3D,E). Consistently, qRT-PCR analysis showed that EFNA5 markedly downregulated the expression of the pro-apoptotic factor TP53 while upregulating the anti-apoptotic factor BCL2 (Figure 3F). Taken together, these findings indicate that EFNA5 enhances oocyte developmental competence by promoting cumulus expansion, improving the cumulus–oocyte microenvironment, and inhibiting apoptosis. The improved cumulus expansion and reduced apoptosis mediated by EFNA5 collectively establish a functional link to the enhanced blastocyst development observed in this study, underscoring its pivotal role during oocyte maturation.

### 3.4. Effects of EFNA5 on the Transcriptional Profile of Oocytes Matured In Vitro

**Figure 4:**
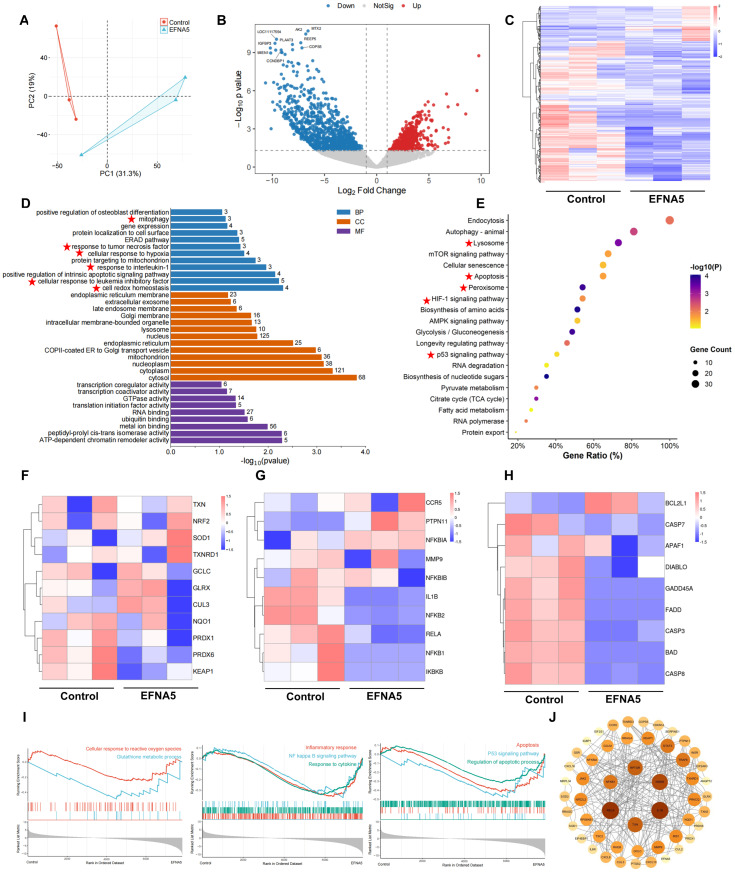
Effects of EFNA5 on the transcriptional profile of oocytes matured in vitro. (**A**) PCA of MII oocyte transcriptomes in control and EFNA5 groups. (**B**) Volcano plot of DEGs; dashed lines indicate |log_2_FC| = 1.5 and *p*-value = 0.05. Red, upregulated; blue, downregulated; grey, not significant. (**C**) Heatmap of DEGs across samples with hierarchical clustering (row-scaled Z-scores). (**D**) GO enrichment (BP/CC/MF); bar length denotes-log_10_(P), numbers indicate gene counts. Highlighted pathways are mainly associated with inflammation and antioxidant defense. (**E**) KEGG pathway enrichment; bubble size represents gene count, color indicates-log_10_(P), *x*-axis shows Gene Ratio. Highlighted pathways are mainly associated with inflammation and antioxidant defense. (**F**–**H**) Module heatmaps for antioxidant (NRF2 targets), inflammatory (NF-κB axis), and apoptotic genes (row-scaled Z-scores). (**I**) GSEA enrichment plots for Glutathione metabolism, NF-κB signaling, and p53 pathway. (**J**) PPI network of core DEGs with hub nodes (node size = degree; edge thickness = interaction confidence). Abbreviations: DEG, differentially expressed gene; BP/CC/MF, biological process/cellular component/molecular function.

To elucidate the molecular mechanisms underlying the beneficial effects of EFNA5 on oocyte developmental competence, we performed RNA-sequencing on MII oocytes from the control and EFNA5-treated groups. Principal-component analysis (PCA) revealed discrete clustering of the two groups, underscoring a robust treatment-specific transcriptomic signature (Figure 4A). Volcano-plot filtering identified 565 differentially expressed genes (DEGs): 129 upregulated and 436 downregulated genes in EFNA5-treated oocytes (Figure 4B). Hierarchical heatmap visualization further highlighted the global transcriptional alterations induced by EFNA5 supplementation (Figure 4C).

GO enrichment analysis demonstrated that these DEGs were predominantly associated with biological processes related to redox homeostasis, regulation of apoptosis, hypoxia response, and cytokine signaling (Figure 4D). Consistently, KEGG pathway analysis revealed significant enrichment in autophagy, apoptosis, lysosome, AMPK and mTOR signaling, HIF-1 signaling, and the p53 pathway, all of which are crucial for stress response and cell survival (Figure 4E). Further inspection of representative genes showed that EFNA5 treatment markedly upregulated classical NRF2 targets such as *SOD2*, while the negative regulators of NRF2 activity, including *KEAP1*, were downregulated, supporting the enhanced activation of the NRF2 pathway (Figure 4F). Conversely, key components of the NF-κB signaling cascade, such as *RELA*, *IKBKB*, and *NFKB1*, were significantly reduced, whereas the inhibitory subunit *NFKBIB* was elevated, indicating effective suppression of NF-κB nuclear activity (Figure 4G). Moreover, apoptosis-related analysis revealed suppression of pro-apoptotic genes such as *BAD*, *CASP3*, *CASP8*, *FADD* and concomitant upregulation of the anti-apoptotic factor *BCL2*, indicating reduced apoptotic activity in EFNA5-treated oocytes (Figure 4H).

GSEA revealed that EFNA5-treated oocytes exhibited significant enrichment of pathways associated with glutathione metabolism, inflammatory signaling, and apoptosis (Figure 4I). Notably, glutathione metabolism was positively enriched, while ROS response, NF-κB signaling, and p53-mediated apoptosis were negatively enriched, reflecting enhanced antioxidant defense, reduced inflammatory activity, and suppression of apoptosis. Protein–protein interaction (PPI) network analysis identified *NFE2L2* (*NRF2*), *RELA*, *IL6*, and *TP53* as hub genes, underscoring their pivotal roles in orchestrating oxidative stress response, apoptosis, and inflammation (Figure 4J). Collectively, these findings demonstrate that EFNA5 supplementation during IVM reprograms the transcriptomic landscape of oocytes, thereby enhancing antioxidant capacity, suppressing inflammatory signaling, and mitigating apoptosis, likely through modulation of the NRF2 and NF-κB pathways.

### 3.5. EFNA5 Enhances Oocyte Redox Homeostasis

**Figure 5:**
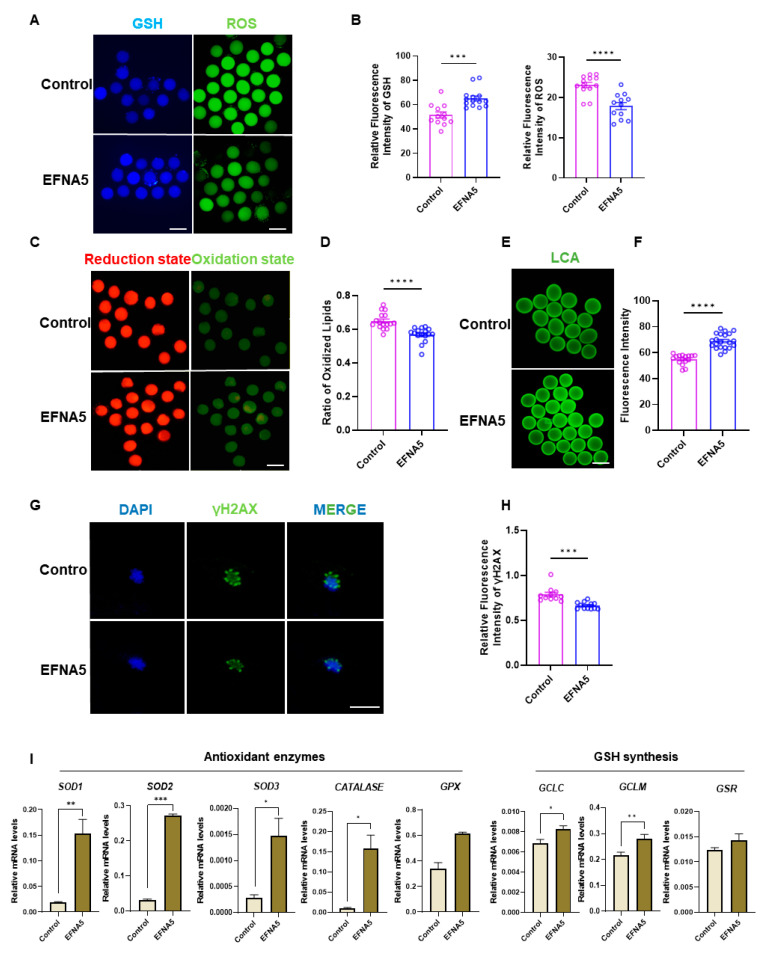
EFNA5 improves oocyte redox homeostasis and oocyte quality. (**A**) Representative fluorescence images of intracellular GSH (left) and ROS (right) in control versus EFNA5-treated oocytes. Scale bar = 50 µm. (**B**) Quantification of relative fluorescence intensity for GSH (left) and ROS (right). (**C**) C11-BODIPY 581/591 labeling of lipid peroxidation showing reduced (red) and oxidized (green) states. Scale bar = 50 µm. (**D**) Ratio of oxidized/reduced C11-BODIPY signals. (**E**) LCA lectin staining of cortical granules in MII oocytes. Scale bar = 50 µm. (**F**) Quantification of LCA fluorescence intensity. (**G**) Immunofluorescence of γH2AX (green) indicating DNA damage (DAPI, blue). Scale bar = 20 µm. (**H**) Quantification of relative γH2AX fluorescence intensity. (**I**) qPCR analysis of antioxidant enzyme genes (*SOD1*, *SOD2*, *SOD3*, *CATALASE*, *GPX*) and GSH synthesis/regeneration genes (*GCLC*, *GCLM*, *GSR*) in Control versus EFNA5-treated oocytes. Abbreviations: GSH, glutathione; ROS, reactive oxygen species; LCA, Lens culinaris agglutinin; γH2AX, phospho-H2AX (Ser139). All experiments were repeated at least three times. Data are shown as mean ± SEM. Statistical significance: * *p* < 0.05, ** *p* < 0.01, *** *p* < 0.001, **** *p* < 0.0001.

To test whether the transcriptome-predicted antioxidant effect of EFNA5 manifests at the cellular level, we profiled redox status and cytological hallmarks of oocyte quality in parallel. EFNA5 markedly lowered intracellular ROS while increasing GSH content (Figure 5A,B). Considering the close relationship between lipid metabolism and oxidative stress [29], we next examined lipid peroxidation accumulation using BODIPY staining. Consistently, lipid peroxidation was attenuated, as evidenced by a reduced oxidized C11-BODIPY 581/591 signal and a decreased oxidized/reduced ratio (Figure 5C,D). At the transcriptional level, EFNA5 upregulated canonical antioxidant genes, including *SOD1*, *SOD2*, *SOD3*, *CATALASE*, *GCLM*, and *GCLC* (Figure 5I). Concomitantly, DNA damage was alleviated, with γH2AX fluorescence significantly reduced (Figure 5G,H), and cytoplasmic maturation indices improved, as shown by stronger and more uniform cortical-granule labeling with LCA lectin (Figure 5E,F). Together, these data indicate that EFNA5 restores redox balance, limits oxidative stress-associated damage, and enhances oocyte quality, thereby supporting higher developmental competence.

### 3.6. EFNA5 Alters the Transcriptional Profile of Cumulus Cells

**Figure 6:**
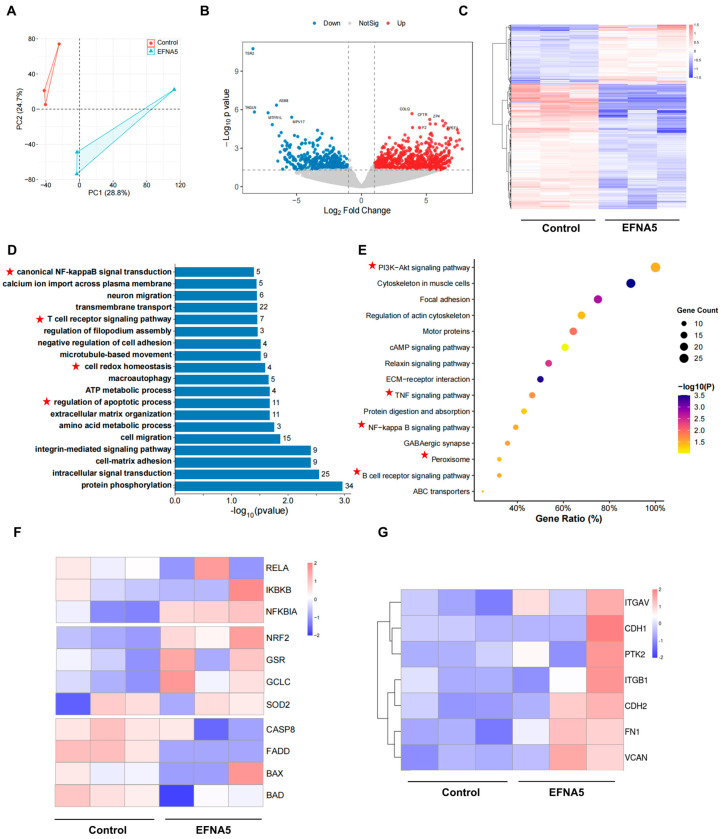
EFNA5 reprograms cumulus cell transcriptomes. (**A**) PCA of RNA-seq profiles from control vs. EFNA5-treated cumulus cells. (**B**) Volcano plot of DEGs; dashed lines denote thresholds |log_2_FC| = 1.5 and *p*-value = 0.05. Red: upregulated; blue: downregulated; gray: not significant. (**C**) Heatmap of DEGs across samples with hierarchical clustering (row-scaled Z-scores; columns: biological replicates). (**D**) Gene Ontology enrichment (BP/CC/MF). Bars indicate-log_10_(P); numbers at bar ends show gene counts. Highlighted pathways are mainly associated with inflammation and antioxidant defense. (**E**) KEGG pathway enrichment. Bubble size represents gene count; color encodes-log_10_(P); *x*-axis shows Gene Ratio. Highlighted pathways are mainly associated with inflammation and antioxidant defense. (**F**) Module heatmap highlighting key pathways in cumulus cells: NF-κB axis (*RELA*, *IKBKB*, *NFKBIA*), NRF2 antioxidant genes (*NRF2*, *GSR*, *GCLC*, *SOD2*), and apoptosis genes (*CASP8*, *FADD*, *BAX*, *BAD*). (**G**) Adhesion-related gene heatmap (row-scaled Z-scores), including *ITGAV*, *ITGB1*, *CDH1*, *CDH2*, *PTK2*, *FN1*, *VCAN*.

To determine how EFNA5 reshapes cumulus cell function, we performed RNA-seq on cumulus cells recovered from COCs matured with or without EFNA5. PCA revealed a clear separation between the two groups, indicating that EFNA5 treatment substantially altered the transcriptomic landscape of cumulus cells (Figure 6A). Volcano plot filtering identified 444 DEGs, including 348 up-regulated and 96 down-regulated genes (Figure 6B). Heatmap visualization further highlighted the EFNA5-induced transcriptional changes, suggesting a broad regulatory impact on cumulus cells (Figure 6C). Functional enrichment of DEGs in EFNA5-treated cumulus cells mirrored the trends observed in oocytes. GO terms were significantly enriched for cellular redox homeostasis and regulation of apoptotic process, together with modules linked to extracellular matrix (ECM) organization, cell–matrix adhesion/integrin-mediated signaling, cytoskeleton dynamics, and protein phosphorylation (Figure 6D). KEGG analysis highlighted PI3K-Akt, TNF and NF-κB signaling pathways (inflammatory control), peroxisome (oxidative detoxification), and adhesion/cytoskeleton pathways including ECM adhesion and associated cytoskeletal remodeling; ABC transporters also emerged among enriched terms (Figure 6E).

Detailed interrogation of key functional pathways revealed that antioxidant genes such as *KEAP1*, *GSR*, *GCLC* and *SOD2* were significantly up-regulated in EFNA5-treated cumulus cells, suggesting activation of the NRF2 pathway (Figure 6F). In contrast, pro-inflammatory genes including *RELA* were down-regulated, indicating an anti-inflammatory effect (Figure 6F). Apoptosis-focused analysis further showed marked down-regulation of pro-apoptotic regulators (*CASP8*, *FADD*, *BAD*), confirming that EFNA5 concurrently dampens oxidative stress, quells inflammation, and curbs apoptosis in cumulus granulosa cells (Figure 6F). This data was concordant with the observed phenotype of reduced apoptosis following EFNA5 treatment. Considering that EFNA5 itself is involved in cell adhesion, we further analyzed cumulus cell adhesion-related genes. The results showed significant upregulation of *ITGB1*, *VCAM1*, *FN1*, and *CDH2* in the EFNA5-treated group (Figure 6G), suggesting that EFNA5 may enhance cumulus cell adhesion and motility, thereby strengthening cumulus cell–oocyte interactions and synergistically supporting oocyte maturation.

### 3.7. EFNA5 Regulates NRF2 and NF-κB Signaling in COCs

**Figure 7:**
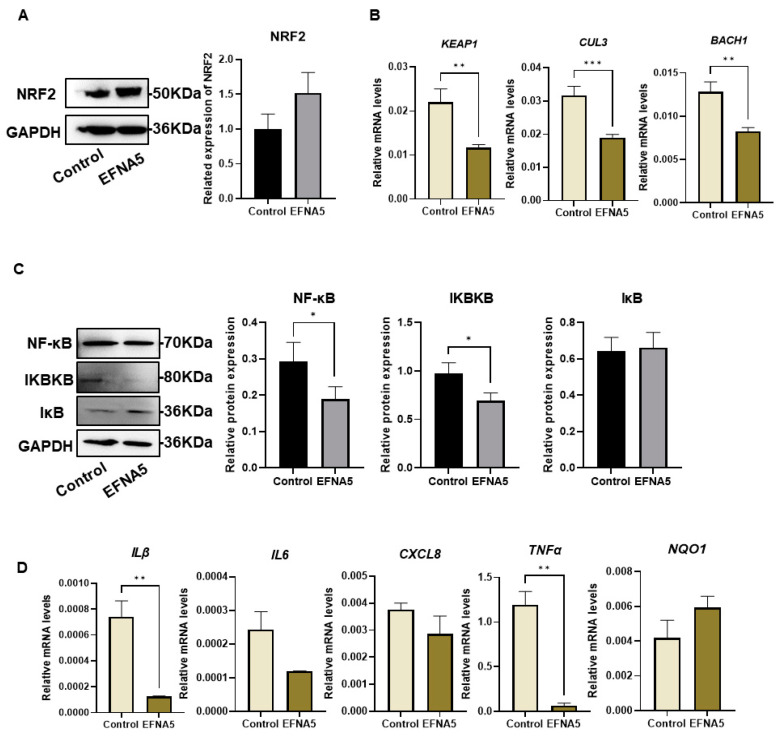
EFNA5 activates NRF2 signaling and suppresses NF-κB activity in COCs. (**A**) Western blot and densitometry of NRF2 in control and EFNA5 groups. Note: The blot image was horizontally flipped to align with the sample order described in the legend. (**B**) Relative mRNA levels of *KEAP1*, *CUL3*, and *BACH1* in control and EFNA5 groups. (**C**) Western blots and densitometry of NF-κB (p65), IKBKB, and IκB. (**D**) Relative mRNA levels of *IL1β*, *IL6*, *CXCL8*, *NQO1*, and *TNFα* in control and EFNA5 groups. All experiments were repeated at least three times. Data are shown as mean ± SEM. Statistical significance: * *p* < 0.05, ** *p* < 0.01, *** *p* < 0.001.

To further elucidate the molecular mechanisms by which EFNA5 enhances the developmental potential of oocytes and the function of cumulus cells, we focused on the NRF2-mediated antioxidant pathway and the NF-κB inflammatory cascade highlighted by our transcriptomic analysis. Western blotting revealed that EFNA5 treatment markedly increased accumulation of NRF2 in oocytes (Figure 7A), suggesting enhanced NRF2 activation. Consistently, the mRNA levels of NRF2 inhibitory regulators, including *KEAP1*, *CUL3*, and *BACH1*, were significantly reduced (Figure 7B), indicating that EFNA5 may potentiate NRF2 activity by suppressing its negative regulators. In parallel, EFNA5 treatment led to a pronounced decrease in phosphorylated NF-κB p65 (p-p65) and IKBKB expression, while IκB levels were slightly elevated (Figure 7C), implying that NF-κB activation was impaired. Consistent with attenuation of NF-κB signaling, EFNA5 significantly reduced the mRNA abundance of *IL1β* and *TNFα* in oocytes (Figure 7D). In contrast, the NRF2 target *NQO1* was upregulated, supporting activation of the antioxidant program. Taken together with the redox results, these data indicate that EFNA5 enhances oocyte quality and developmental competence by activating NRF2-driven antioxidant defenses while concurrently dampening NF-κB-dependent pro-inflammatory cytokine transcription.

**Figure 8:**
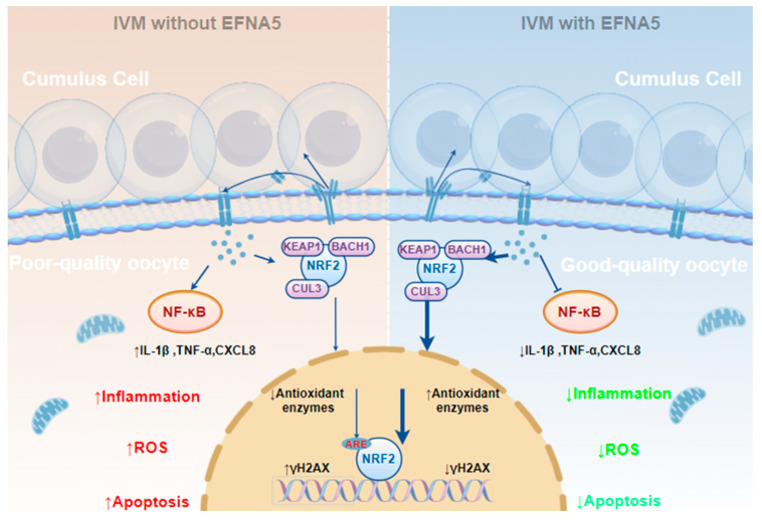
Working model of EFNA5 action in promoting oocyte developmental competence. Left, IVM without EFNA5; right, IVM with 50 ng/mL EFNA5. In cumulus cells and the oocyte, EFNA5 attenuates NF-κB signaling and its cytokine outputs (*IL-6*, *TNFα*, *CXCL8*), while promoting release of NRF2 from the KEAP1-CUL3-BACH1 inhibitory complex, nuclear translocation of NRF2, and transcription of antioxidant-related downstream genes (*GSH*, *SOD*, *GCLM/GCLC*, *CATALASE*). Solid arrows indicate activation or positive regulation, blunt-ended lines represent inhibition or suppression, and upward or downward arrows indicate increased or decreased levels, respectively. The figure was generated using Figdraw (www.figdraw.com).

Based on these findings, we propose a working model of EFNA5 action (Figure 8): EFNA5 activates the NRF2 antioxidant axis by downregulating its inhibitory regulators (*KEAP1*, *CUL3*, and *BACH1*) and upregulating the target gene *NQO1*, while concomitantly restraining NF-κB signaling. This dual action reduces transcription of the pro-inflammatory cytokines *IL1β* and *TNFα* and mitigates oxidative stress, thereby improving oocyte developmental competence.

## 4. Discussion

IVM is a critical step in mammalian ART, yet the limited maturation quality and developmental competence of oocytes remain major obstacles to its broader application [30,31]. ODFs play central roles in maintaining oocyte developmental potential and shaping the follicular microenvironment. Among them, the classical ODFs, GDF9 and BMP15, have been shown to regulate cumulus cell proliferation and extracellular matrix formation, thereby supporting folliculogenesis and embryo development [31,32]. However, the diversity and functional mechanisms of ODFs remain incompletely understood. In this study, we provide the first systematic evidence that EFNA5 functions as a bona fide oocyte-derived factor (ODF) that orchestrates IVM competence at both the oocyte and cumulus levels. Mechanistically, EFNA5 enhances the NRF2-mediated antioxidant defense pathway while concurrently suppressing NF-κB-driven inflammatory signaling, thereby attenuating ROS accumulation, reducing apoptosis, and stabilizing redox homeostasis. These coordinated effects ultimately improve oocyte quality and developmental competence under in vitro maturation conditions.

Cross-species transcriptomic analyses revealed that EFNA5 is stably expressed in oocytes, indicating its evolutionary conservation as a ODF. In the ovine model, EFNA5 was predominantly enriched in oocytes, whereas its main receptor, EPHA4, showed expression both in oocytes and granulosa cells. This spatiotemporal specificity suggests that EFNA5 may act via autocrine or paracrine signaling to orchestrate oocyte maturation, providing a theoretical basis for its functional study in vitro. Notably, both the direction and magnitude of EFNA5’s effects appear to be species dependent. In human and sheep, EFNA5 abundance is lower in IVM than in in vivo-matured oocytes, whereas mice show the opposite trend. EFNA5 has been reported to promote apoptosis in murine granulosa cells [17], yet in sheep it exerts a clearly anti-apoptotic action that protects oocytes and improves embryo quality.

A straightforward explanation is that EFNA5 engages different EphA receptors across species, and the abundance of these receptors varies between oocytes and granulosa cells. EFNA5 can bind to multiple EPA isoforms, including EPHA4, EPHA5, and EPHA7, but the relative receptor expression differs not only across species but also between cell types within the same species [33,34,35]. Distinct EphA receptors recruit different adaptor protein complexes and preferentially activate specific downstream pathways. For instance, in pancreatic islet cells, EFNA5 interacts with EPHA5 to mediate intercellular communication and regulate glucose-stimulated insulin secretion [36]. In neuronal and epithelial cells, EFNA5 binding to EPHA3 or EPHA7 modulates cell adhesion and cytoskeletal organization [37]. In our ovine dataset, EPHA4 was identified as the predominant receptor in granulosa cells, whereas in the mouse ovary, other EphA isoforms appear to be more enriched [17]. In addition, species-specific differences have been widely reported for other ODFs. In mice, GDF9 and BMP15 strongly promote cumulus expansion and enhance oocyte developmental competence [30]. In our experiments, EFNA5 can promote cumulus expansion, reduce apoptosis, and improve blastocyst quality, which conceptually aligns with previous studies on classical oocyte-secreted factors. Notably, GDF9 deficiency leads to complete follicular arrest, whereas BMP15 deficiency results only in reduced fertilization efficiency and subfertility [38]. By contrast, in sheep, both GDF9 and BMP15 are indispensable; immune neutralization of either factor causes follicular blockage and anovulation in most ewes, while partial neutralization of BMP15 markedly increases ovulation rate, suggesting a dose-dependent effect [32]. In cattle, GDF9 and BMP15 play indispensable roles in follicular development and the ovulatory process [39], while in humans, mutations or deficiencies in these factors are more often associated with diminished ovarian reserve or subfertility rather than absolute infertility [40,41]. Collectively, these findings highlight that ODF-mediated mechanisms exhibit pronounced interspecies differences in both functional significance and regulatory strength.

Using the ovine IVM model, we further elucidated the molecular actions of EFNA5. Supplementation with 50 ng/mL EFNA5 significantly increased blastocyst rates, with enhanced total cell numbers and reduced cell apoptosis. These findings indicate that EFNA5 may not only promote cell proliferation but also mitigate apoptosis, thereby improving overall blastocyst quality. Such effects align with previous studies demonstrating that OFDs, including GDF9 and BMP15, regulate granulosa cell proliferation, extracellular matrix formation, and key signaling pathways to support follicle growth, ovulation, and early embryonic development [31,32]. Furthermore, the expansion of COCs serves as an important indicator of granulosa cell function and oocyte competence during IVM [42]. COCs’ expansion reflects granulosa cell secretion of hyaluronic acid and remodeling of the extracellular matrix, processes that provide nutritional and signaling support to oocytes and maintain microenvironmental homeostasis [21]. EFNA5 supplementation markedly increased COCs expansion and reduced oocyte apoptosis, suggesting that EFNA5 enhances oocyte–granulosa cell interactions and protects oocytes from apoptotic damage. Notably, EFNA5 exhibited an optimal concentration effect, with 50 ng/mL being most effective for improving oocyte maturation and quality, while higher doses resulted in attenuated benefits, possibly due to feedback inhibition by endogenous EFNA5. Oxidative stress and inflammation are major limiting factors for oocyte developmental competence during IVM [43,44]. IVM oocytes frequently exhibit elevated ROS levels, depleted glutathione, and lipid peroxidation accumulation, which disrupt intracellular redox homeostasis, increase DNA damage, and cause spindle abnormalities, ultimately restricting oocyte maturation and subsequent embryonic development [45]. Activation of inflammatory pathways can further induce pro-apoptotic gene expression, exacerbating oocyte damage [46]. In this study, EFNA5 treatment significantly upregulated NRF2-mediated antioxidant pathways, including SOD2 and GPX4, while suppressing NF-κB signaling and downstream pro-inflammatory genes such as IL6 and TNFα. This dual regulation of oxidative stress and inflammation likely contributes to the improved oocyte quality and enhanced embryonic potential observed in EFNA5-treated groups. The role of EFNA5 in modulating such responses is further corroborated by complementary studies in neuronal, endothelial, and metabolic tissues [47,48].

Cell adhesion is essential for intercellular communication, microenvironmental stability, and tissue integrity, particularly in cumulus cells surrounding oocytes. Strong adhesion maintains the structural integrity of the COCs, which is critical for oocyte maturation [49,50]. EFNA5, as a membrane-bound ligand interacting with EphA receptors, mediates contact-dependent signaling that regulates cytoskeletal remodeling, strengthens intercellular adhesion, and stabilizes cell–matrix attachments. In neurons, EFNA5 modulates adhesion proteins such as integrins and focal adhesion components to guide axon pathfinding and synaptic stability; in endothelial cells, it promotes cell–cell junctions and vascular formation [51,52]. Our granulosa cell transcriptome analysis suggests that EFNA5 may enhance adhesion-related gene expression, indicating a potential mechanism for improved COC stability and oocyte support, although functional validation of key adhesion molecules is warranted.

Here, we provide the first comprehensive evidence establishing EFNA5 as a newly identified oocyte-derived factor that significantly improves IVM performance. EFNA5 improves antioxidant capacity, suppresses inflammatory signaling, reduces apoptosis, and potentially regulates cell adhesion and motility, thereby optimizing the oocyte microenvironment and maintaining blastocyst quality. In conclusion, EFNA5 provides a novel molecular foundation for optimizing IVM systems and offers critical insights into the function of oocyte-derived factors. Further elucidation of the synergistic effects of multiple oocyte-derived factors could pave the way for enhancing mammalian oocyte developmental competence and refining clinical ART strategies.

## 5. Conclusions

EFNA5 emerges as an evolutionarily conserved oocyte-derived ligand that safeguards oocyte competence during in vitro maturation. By activating NRF2-driven antioxidant defenses and dampening NF-κB-mediated inflammation, EFNA5 enhances cumulus–oocyte communication, lowers apoptosis, and improves cytoplasmic and nuclear maturation. Functionally, EFNA5 supplementation increases blastocyst formation and embryo quality. These findings establish EFNA5 as a novel oocyte-secreted regulator essential for protecting oocyte competence under culture-induced stress and highlight its potential application in optimizing IVM systems and ART.

## Data Availability

The data are available from the first author, Xingyuan Liu (19806395675@163.com), upon request.

## Supplementary Materials

The following supporting information can be downloaded at: https://www.mdpi.com/article/10.3390/antiox14121476/s1.
